# Spectacles

**Published:** 1876-07

**Authors:** 


The Bistoury.

ELMIRA, N. Y., JULY, 1876.

The BISTOURY is published Quarterly, upon the 1st of April, July, October and January, at Fifty Cents a year, in advance.

### SPECTACLES.

Having been consulted recently by an unusual number of persons requiring aids to vision in the form of spectacles, we are surprised to find how many persons are injuring their eyes with illy-contrived and badly adjusted glasses. Not only do we find persons wearing spectacles who do not require them,—so doing an incalculable injury to their eyes;—but also those who have made their own selection of lenses, and used the most reckless judgment in so doing. It is not unfrequently the case that we find parties wearing glasses eight or ten numbers too strong for them, assigning as a reason for so doing, that it rendered printing larger for them, or that it was the best they could do where they made the purchase. Then, we have found illy-fitted lenses, held in awkward and badly contrived frames, with one lens of two or three numbers stronger than its mate, held in frames that would not keep both lenses opposite each eye at the same time, producing, often, the most serious consequences.

People should learn two things with reference to the selection of glasses, if they would preserve their vision unimpaired to the latest period of life:

First. That it is necessary and always safer to apply to an ophthalmic surgeon and have their eyes accurately and properly fitted to lenses of the style and power that is precisely adapted to the peculiar condition of their eyes. The ophthalmic surgeon, after testing an eye, gives a formula, in which he prescribes the necessary lens to be worn over each eye, when the patient applies to the optician or spectacle dealer and then makes his purchase intelligently. Great care must be exercised that the spectacles fit accurately, in that the glasses are on the same level, so that one is not higher than its fellow, that the centre of the lens is precisely over the centre of the pupil, and that the lenses are sufficiently close to the eye. In order to secure the last mentioned object, neatly made frames are necessary, frames that will fit the nose and curl behind the ears, in order to hold the lenses firmly and evenly before the eyes.

Secondly. A great difference exists in the various makes of spectacles. Many kinds should not be purchased at all,—or at any price. These are the imperfectly ground lenses, with cheap, blue frames, that will fit no nose of modern construction, nor will the lenses permit a ray of light to pass in the direction intended. They are sold to dealers at from two to three dollars a dozen, and retailed to the unsuspecting and over credulous, at one and a half or two dollars a pair. Such glasses are worse than useless,—they are absolutely harmful.

We have occasionally met with persons whose eyes were caused to squint by reason of the glasses not resting evenly upon the nose, and so bringing the centre of the lens opposite the pupil.

When concave glasses are worn, to correct near-sightedness, they should always be placed as near the eyes as possible, otherwise the size of the object will be diminished and the retinal picture rendered indistinct. Convex lenses, those worn for "old-sightedness," should be worn so that the spectacle frame lodges near the centre of the nose, setting evenly and firmly in that position. This arrangement has a tendency of rendering the retinal image larger and more distinct. An eye glass,—such as are worn by fops, there being but one glass, through which they peep with one eye, should never be used. It is apt to cause a weakness in one eye for lack of proper use.

The very best glasses that can be procured are those that are termed *periscopic*, their peculiarity being that they are ground convex on one side and concave on the other, so as to avoid much of the spherical aberration of light. They should be slightly violet tinted, which will reject the caloric or heat rays of light, which will be found a great comfort to those who are compelled to wear glasses continually. The best glasses of this character that we have been able to find, and which we can conscientiously recommend, are made by T. A. Willson & Co., of Reading, Pennsylvania. Their lenses are not only accurately, evenly, and beautifully ground and polished, but are likewise perfectly tinted, and are mounted in the neatest and most perfectly adapted frames we have ever seen. It is so rare or never the case that we encounter all these excellencies combined, in spectacles, that it becomes a real pleasure to make this public endorsement of these lenses, and to recommend them in all cases where spectacles are needed, first remembering, however, to have them suitably fitted by a competent person.

